# The Challenge of Tightening Door-to-Needle Timings in a Telestroke Setting: An Emergency Medicine Driven Initiative

**DOI:** 10.7759/cureus.12316

**Published:** 2020-12-27

**Authors:** Bao Yu Geraldine Leong, Hui Min Joyce Ni, Ling Tiah, Camlyn Tan

**Affiliations:** 1 Emergency Medicine, Changi General Hospital, Singapore, SGP; 2 Performance Improvement, Changi General Hospital, Singapore, SGP

**Keywords:** stroke, telemedicine, dtn, emergency department

## Abstract

Introduction

Administering intravenous thrombolytic therapy within 60 minutes on arrival in any healthcare facility is challenging, especially when done by Emergency Medicine Physicians (EMP) via telemedicine in centres without onsite neurology cover. Prior quality improvement interventions have improved median Door-to-Needle (DTN) timings in our centre; however, it still falls short of the DTN target of 60 minutes.

Methods

Various quality improvement interventions were implemented over four months by a multi-disciplinary telestroke workgroup led by EMPs to improve DTN timings for patients presenting with acute ischaemic strokes. A retrospective observational study was conducted to review if these interventions resulted in an improvement in DTN timings while keeping the rates of stroke mimics given thrombolytic therapy, haemorrhagic conversions and 30-day mortality rates low.

Results

A total of 279 patients were evaluated. Median DTN timings significantly improved from 71.0 minutes pre-intervention to 62.0 minutes post-intervention (p=0.012). Correspondingly, the proportion of patients with DTN ≤ 60 minutes increased from 31.7% pre-intervention to 47.0% post-intervention, giving an odds ratio of 1.91 (95% CI 1.17 - 3.11, p=0.009). There were no significant differences found in the rates of stroke mimics, haemorrhagic conversions and 30-day mortality pre and post-intervention.

Conclusion

The implementation of EMP led to systemic quality improvement interventions is associated with improved DTN timings without compromising clinical quality outcome measures like haemorrhagic conversion rates and 30-day mortality rates. EMPs, with a broad knowledge base and familiarity, interacting with various specialities and co-ordinating care, are uniquely suited in this role to drive change. More work in the public health sector would also have to be done to improve the population’s response to acute stroke symptoms.

## Introduction

In our current day and age, administering intravenous thrombolytic therapy for acute ischaemic stroke in eligible patients presenting to the Emergency Department (ED) in hopes of improving neurological outcomes is considered standard of care [1]. As per the American Heart Association/American Stroke Association (AHA/ASA) guidelines [2], the benefit of thrombolytic therapy is time-dependent and should be initiated as soon as possible. Delayed thrombolytic therapy administration can result in poor clinical outcomes like increased intracranial haemorrhage risks [3,4]. Although it has been recommended that patients with acute ischaemic stroke should receive intravenous thrombolytic therapy within 60 minutes [5] on arrival to any healthcare facility, in reality, many patients do not receive thrombolytic therapy on time [6-9]. As such, decreasing Door-to-Needle (DTN) time (time taken from the arrival of the patient at the ED doors to administrating thrombolytic therapy to the patient) is a major challenge for administering thrombolytic therapy, especially so when it is done by Emergency Medicine Physicians (EMP) via telemedicine (i.e. telestroke) in sites without onsite neurology cover.

Background

**Figure 1:**
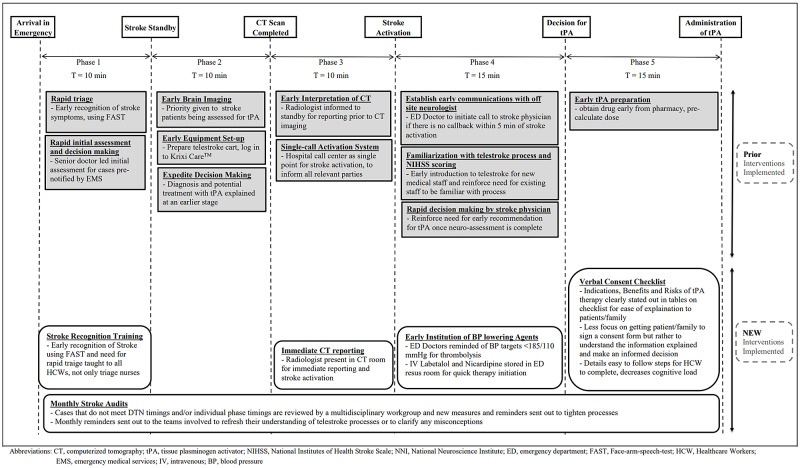
Summary of the five phases of the telestroke process with prior and new quality improvement interventions tPA- tissue plasminogen activator; FAST- Facial drooping, Arm weakness, Speech difficulties and Time; ED- Emergency Department; EMS- Emergency Medical Services; NIHSS- National Institutes of Health Stroke Scale; DTN- Door-to-Needle.

This study was conducted at an urban Emergency Department (ED) with an average ED daily attendance of 420 patients, in a 1,000-bed acute regional hospital without onsite neurology cover where a hub-and-spoke model of telestroke was developed. All screened eligible patients presenting with acute stroke symptoms to the ED (spoke) would undergo an immediate Computer Tomography (CT) scan of the brain before a video-assisted evaluation by off-site Neurologists at a national specialist centre (hub). This web-based video conferencing process was facilitated by using the KrixiCareTM program, which allowed a real-time audio-visual link between both centres and CT images transfer. The entire telestroke process is reliant on the Neurologist, ED staff and Radiology staff to work together efficiently and quickly to achieve the goal of a Door-to-Needle (DTN) timing of 60 minutes or less. As part of prior quality improvement initiatives, the telestroke process had been subdivided into five phases for the ease of analysis and to aid in implementing specific interventions [10,11], with the target timings of the respective phases as indicated in Figure 1.

Prior quality improvement initiatives had seen the median DTN timings drop from an initial of 92.7min to 86min [10] and a subsequent 78 min [11]. However, this was still short of the DTN target of 60min. A multi-disciplinary inter-hospital stroke committee led by EMPs comprising of EMPs, ED Nurses, Neurologists, Radiologists, Radiographers, Stroke Nurse Clinicians and IT support staff was set up to look into this shortfall, resulting in a series of quality improvement interventions that were carried out from June to September 2016 to improve DTN timings further.

Objective

The objective of this study was to evaluate if the various quality improvement interventions implemented from June to September 2016 resulted in an improvement in the DTN timings for patients presenting with acute ischaemic stroke to the ED; while at the same time, ensuring that clinical standards were maintained by keeping the rates of stroke mimics given thrombolytic therapy, haemorrhagic conversions and 30-day mortality rates low.

## Materials and methods

This retrospective observational study was approved by the Centralised Institutional Review Board (CIRB) of the study institution and consent waiver was approved as all data collected were anonymised. All patients aged ≥21 years old who were administered thrombolytic therapy for an acute ischaemic stroke in the ED from January 2015 to December 2017 were included in this study. Patients who were not in the thrombolytic window, had contraindications to thrombolytic therapy, were not offered thrombolytic therapy by the neurologist or declined thrombolytic therapy were excluded from the study. Trained research assistants extracted required data from patient electronic medical records and phone operator call logs. As the series of quality improvement interventions were implemented gradually over time from June to September 2016, the Pre-intervention period would be defined as from January 2015 to August 2016 and the Post-intervention period would be from September 2016 to December 2017. However, during the study CIRB renewal in November 2017, the study consent waiver was no longer approved, resulting in excluding the data collected from November to December 2017 to comply with the new CIRB conditions. Thus, the Post-intervention period would run from September 2016 to October 2017 instead.

This study’s primary outcome measure was the median DTN timings, including the breakdown of timings for each phase, and the proportion of patients achieving the DTN target of 60 minutes. Secondary outcome measures include rates of intracranial haemorrhagic conversion post thrombolysis, rates of stroke mimics given thrombolytic therapy, 30-day mortality rates and Modified Rankin Score (MRS) on discharge from hospital.

Statistical analysis was performed with SPSS statistical software, version 19.0 (IBM Corp. Armonk, NY). Categorical data were presented as frequency (percentage). Numeric data were presented as mean (standard deviation) for parametric distribution and median (interquartile range) for non-parametric distribution. The differences in characteristics between pre-and post-intervention were examined using Chi-Square test or Fisher’s Exact test for categorical variables, two-sample t-test or Mann Whitney U-test continuous variables, where appropriate. Odds Ratio (OR) was reported where appropriate. A two-tailed, p-value of <0.05 was considered statistically significant.

Interventions implemented

Monthly stroke audits, where an in-depth analysis of factors contributing to delays in each of the phases, were carried out by an EMP led multi-disciplinary workgroup to review cases from the previous month that did not meet DTN targets and new measures (as detailed in Figure 1) to tighten the processes were implemented. Monthly reminders were also sent to the managing team members to highlight various processes that might have been overlooked and clarify any misconceptions. Teaching sessions helmed by EMPs and Stroke Nurse Clinicians were also organised to educate all healthcare workers on the early recognition of stroke and the need for early and rapid triage. One of the major causes for a delay that was flagged up during the audits was the time spent to achieve Blood Pressure (BP) targets before thrombolytic therapy administration. ED staff were reminded of the need for early BP control via reminder emails and teaching sessions, and BP-lowering medications were placed in convenient locations for rapid therapy initiation.

Instead of the traditional signed consent form for consent to thrombolytic therapy, a verbal consent checklist was also implemented during this period. In the new verbal consent checklist, steps required for consent taking were all listed down systematically, decreasing the cognitive load on the EMP facing the time pressures of administering thrombolytic therapy. It included information essential to consent taking with the indications, benefits and risks of thrombolytic therapy all clearly stated out in tables with easy to understand percentages. Using this new model, there was less focus on getting patients or their families to sign a generic consent form. Instead, the emphasis was placed on getting the patients and their families to understand the information explained and make an informed decision. All the interventions described have been summarised in Figure 1.

## Results

A total of 279 patients were given thrombolytic therapy during the study period, with 145 in the Pre-intervention group and 134 in the Post-intervention group. The demographics and clinical characteristics of patients in both groups are presented in Table 1. Other than there being more smokers in the Pre-intervention group, there were no significant differences seen in patient baseline characteristics in both groups. More importantly, the median National Institutes of Health Stroke Scale (NIHSS) scores on both groups' presentation were similar, showing no significant difference in the severity of strokes in either group.

**Table 1:** Demographics and clinical characteristics of patients pre- and post-intervention CVA: cerebrovascular accident; IQR: interquartile range; NIHSS: National Institutes of Health Stroke Scale

Baseline characteristics	Pre-intervention (n = 145)	Post-intervention (n = 134)	p-value
Age (mean ± SD)	64.4 ± 13.7	65.9 ± 13.5	0.343
Male (n, %)	94 (64.8%)	88 (65.7%)	0.882
Race (n, %)			0.341
Chinese	87 (60.0%)	87 (64.9%)	
Malay	24 (16.6%)	26 (19.4%)	
Indian	14 (9.7%)	11 (8.2%)	
Others	20 (13.8%)	10 (7.5%)	
Comorbidities (n, %)			
Hypertension	100 (69.0%)	100 (74.6%)	0.294
Diabetes Mellitus	42 (29.0%)	37 (27.6%)	0.802
Ischaemic Heart Disease	63 (43.4%)	57 (42.5%)	0.878
Previous CVA	13 (9.0%)	19 (14.2%)	0.172
Hyperlipidemia	123 (84.8%)	109 (81.3%)	0.437
Atrial Fibrillation	0 (0.0%)	0 (0.0%)	1.000
Impaired Fasting Glucose	0 (0.0%)	1 (0.7%)	1.000
Smoker (n, %)	66/130 (50.8%)	41/116 (30.6%)	0.038
NIHSS score on presentation (median, IQR)	9.0 (5.0-15.0)	7.5 (4.0-15.0)	0.352
Stroke Onset to ED Door, minutes (median, IQR)	60.0 (43.0-110.0)	63.5 (43.0-115.0)	0.656

After the quality improvement interventions were implemented, there was a significant improvement in median DTN timings from 71.0 minutes to 62.0 minutes (p=0.012) as shown in Table 2. Correspondingly, there was also an increase in the proportion of patients with DTN ≤ 60 minutes from 31.7% pre-intervention to 47.0% post-intervention (refer to Table 2), giving an odds ratio 1.91 (95% CI 1.17 - 3.11, p=0.009). The timings obtained for each of the five phases in the telestroke process were also compared and analysed. Phases 2 and 3 were analysed together as the addition of a CT multiphasic angiography to the standard plain CT brain for all patients halfway through our study period made data collection for a time of CT scan completion inhomogeneous. The phase that had the most improvement post-intervention was Phase 5, which represented the period from neurology offer of thrombolytic therapy to the administration of thrombolytic therapy, typically where consent for administration of thrombolytic therapy was taken. The median timing for Phase 5 improved from 21.5 minutes (IQR 12.8 - 30.0 min) pre-intervention to 15.0 minutes (IQR 8.0 - 25.0 min) post-intervention (p=0.002, refer to Table 2).

**Table 2:** Outcomes measures DTN: Door-to-needle; MRS: Modified Rankin Score; IQR: interquartile range; OR: odds ratio; CI: Confidence Interval

Outcome measures	Pre Intervention (n = 145)	Post Intervention (n = 134)	Difference	OR (95% CI)	p-value
Primary Outcome Measure					
DTN, minutes (median, IQR)	71.0 (57.0-86.5)	62.0 (53.0-81.0)	-9.0	-	0.012
Phase 1, minutes (median, IQR)	6.0 (3.0-8.0)	7.5 (4.0-12.0)	+1.5	-	0.003
Composite Phase 2 & 3, minutes (median, IQR)	15.0 (12.0-19.0)	13.0 (11.0-17.0)	-2.0	-	0.002
Phase 4, minutes (median, IQR)	21.0 (19.0-28.0)	22.0 (17.0-27.0)	+1.0	-	0.518
Phase 5, minutes (median, IQR)	21.5 (12.8-30.0)	15.0 (8.0-25.0)	-6.5	-	0.002
DTN ≤ 60 min (event rate, percentage)	46 (31.7%)	63 (47.0%)	+15.3%	1.91 (1.17-3.11)	0.009
Secondary Outcome Measures					
MRS score (mean ± SD)	2.86 ± 1.46 (n=118)	2.96 ± 1.63 (n=94)	-	-	0.820
Haemorrhagic conversion (event rate, percentage)	25 (17.2%)	24 (17.9%)	+0.7%	1.05 (0.57-1.94)	0.883
Stroke mimic (event rate, percentage)	9 (6.2%)	8 (6.0%)	-0.2%	0.96 (0.36-2.56)	0.934
30-day mortality (event rate, percentage)	4 (2.8%)	4 (3.0%)	+0.2%	1.09 (0.27-4.43)	0.910

The intracranial haemorrhagic conversion rates post thrombolytic therapy pre-, and post-intervention remained almost similar and had a calculated odds ratio of 1.05 (95% CI 0.57 - 1.94). The numbers of stroke mimics also remained almost similar to pre-and post-intervention with a calculated odds ratio of 0.96 (95% CI 0.36 - 2.56). Both secondary outcomes were found not to be statistically significant (refer to Table 2). Analysis of the Modified Rankin Scores of patients on discharge during the study period gave a mean score of 2.86 ± 1.46 pre-intervention and a mean score of 2.96 ± 1.63 post-intervention, which was found to be not statistically significant as well (refer to Table 2). Likewise, as shown in Table 2, the difference in 30-day mortality rates pre-and post-intervention was also not statistically significant with a calculated odds ratio of 1.09 (95% CI 0.27 - 4.43).

## Discussion

The systemic quality improvement interventions described have helped significantly reduce DTN timings, as shown in the results above. Although the ultimate goal of all eligible patients receiving thrombolytic therapy within 60 minutes was not achieved, a significant improvement in the proportion of patients achieving this goal from 31.7% to 41% was shown after the implementation of our methods. In truth, telestroke involves extra hurdles and multiple stakeholders like ED, radiology, neurology and even IT support to work closely together. Thus, it is conceivable that DTN timings would be longer than that for direct stroke activations where the in-house neurology team takes over direct management of the acute stroke patient. Other telestroke centres have also reported comparable DTN timings of 61-106 minutes [8,12-15], further illustrating the challenge to achieve DTN timings of less than 60 minutes in such settings.

The intention of dividing into five phases, as described above is to break down the large, complicated telestroke process into five small time capsules that are more easily understood and analysed. Within each of the five phases, the telestroke workgroup led by EMPs and comprising of all the vital stakeholders - EMPs and ED Nurses, Neurologists, Radiologists, Radiographers and IT support - brainstormed and came up with quality improvement interventions to smoothen the telestroke process out (refer to Figure 1). In particular, the monthly stroke audit, where cases that do not meet DTN timings are brought up and discussed by a multi-disciplinary workgroup comprising all the stakeholders, were particularly useful in refining processes clarifying misconceptions. These discussions provide a suitable feedback mechanism for various stakeholders in the telestroke process on what works on the ground and what needs to be fixed on the ground for the telestroke process to run smoothly.

Comparing with other described quality improvement interventions in the literature [16-18], most prominently of which is the Target: Stroke series of initiatives put together by the AHA/ASA [16], there were some similar process changes described like implementing a team-based approach and rapid feedback to stakeholders/stroke team on their performance about DTN timing issues. However, there are two unique features in the above-described quality improvement initiatives that have not been highlighted previously - early initiation of BP lower agents and the use of a verbal consent checklist. As described above, EMPs were reminded of the BP targets for thrombolysis and the need for early BP control, and logistically, BP-lowering medications were placed in convenient locations for rapid therapy initiation. This intervention allowed BP targets to be achieved earlier and thus, thrombolysis to be administered earlier and the resultant DTN to be reduced. The benefits of using a verbal consent checklist are further elaborated below. Another interesting feature is that the quality improvement initiatives described above are mostly EMP led. This differs from the largely by neurologist led workflows or processes that have been described in the literature [16-18]. EMPs have the advantage of having a broad knowledge base, familiarity interacting with various specialities and coordinating care, and, most importantly of all, familiarity with the workflows and logistical requirements in the ED where most of the DTN processes take place. These factors make EMPs uniquely suited for a leading role to drive change in this particular setting. EMPs should be encouraged to take up this challenge in their various institutions.

Looking at the analysis of the timings obtained for the different phases, the results show a significant increase in the timings obtained for Phase 1, which describes the period starting from patient ED arrival to a stroke standby being called (refer to Figure 1). The median timing was 6.0 minutes pre-intervention and increased to 7.5 minutes post-intervention (refer to Table 2). Improving triage timings and stroke recognition by non-physician staff has always been a challenge in many emergency departments. The workgroup had previously identified this issue, and Stroke Recognition Training had been implemented. EMPs and Stroke Nurse Clinicians teach all healthcare workers the recognition and treatment of patients presenting with acute stroke symptoms. However, this training was previously held only once every four to six months on an ad hoc basis and coupled with the high turnover rates of ED frontline staff; there has not been much positive impact seen. Moving forward, the number of scheduled training sessions would have to be increased, and an EMP has been appointed to oversee this process.

On the other hand, Phase 5, which describes the period from neurology offer of thrombolytic therapy to the administration of thrombolytic therapy showed the most improvement - from a median of 21.5 minutes pre-intervention to 15.0 minutes post-intervention (refer to Table 2). This can be largely attributed to the introduction of a Verbal Consent Checklist where the indications, benefits and risks of thrombolytic therapy are clearly stated out in straightforward and simple language - making it much easier, especially for junior ED staff and non-specialist ED staff, to help patients and their families make an informed decision. This is especially useful. It helps decrease the care provider's cognitive load. It guides the care provider on necessary informed consent taking steps, making it more structured and straight forward and ultimately helping the patients and their families come to an informed decision more quickly. It is recommended that all centres use a similar checklist to help facilitate essential emergent informed consent taking.

The secondary outcome measures, the haemorrhagic conversion rates, stroke mimic rates, MRS scores and 30-day mortality rates have remained largely similar pre and post-intervention despite significant improvement in DTN timings and the proportion of patients achieving target DTN timings. This finding was unexpected, as improved clinical outcomes were expected to improve DTN timings [1,4,16] significantly. This might be due to the relatively small sample size, which was not powered to detect any differences. Also, further analysis of the data revealed that although there was a significant decrease in DTN timings, the median time taken from stroke onset to patient arriving at ED (Stroke onset to ED door) remained largely the same - at 60.0 minutes pre-intervention and 63.5 minutes post-intervention (p=0.656), as shown in Table 1. With measured acute stroke presentation at ED (Stroke onset to ED door) timings longer than DTN target timings, it illustrates the fact that the patient population continues to present late to the Emergency Department for acute stroke symptoms - which may be a reason why there were no significant improvements seen in the clinical outcomes despite significant improvement in DTN timings. To address this issue, public health measures should be implemented across the nation to educate the general public on identifying signs and symptoms of a stroke and seeking medical help early for stroke. 

Limitations

As a retrospective observational study, our results are inherently affected by the accuracy of available documented records and recall bias. Also, given its retrospective nature, our study would not establish any causal relationships from the interventions done. Any confounders and biases would also not be able to be controlled. However, as the focus of this study was to share our experience of identifying areas for improvement and systemic quality improvement interventions implemented helped improve DTN, we feel that the above limitations do not detract too much from the original goals of the study.

Also, specific clinical outcomes like 30-day NIHSS scores, three-month MRS scores and discharge destinations were not captured. Our primary focus was on the impact these new systemic quality improvement interventions had on DTN timings. The sample size was also not pre-calculated to detect significant differences in mortality rates. Lastly, our study is a single-centre study and the interventions and results described may not be as applicable to other patient populations and settings.

## Conclusions

In conclusion, the implementation of systemic quality improvement initiatives as detailed above with the buy-in of all the stakeholders involved is shown to be associated with improved DTN timings for patients receiving thrombolytic therapy for acute ischaemic stroke. Our median DTN timing post-intervention at 62min, though not the gold standard, came very close to the ideal target of 60 minutes recommended by AHA/ASA. Although telestroke quality improvement interventions have improved DTN timings, clinical quality outcome measures like the haemorrhagic conversion rates and 30-day mortality rates have remained largely stable. EMPs, with a broad knowledge base and familiarity, interacting with various specialities and coordinating care, are uniquely suited in this role to drive change. More work in the public health sector would have to be done to improve the population's response to acute stroke symptoms.
